# Constrained RNA polymerase mutational pathways distinguish corallopyronin A resistance from rifampicin resistance in staphylococci

**DOI:** 10.1038/s41598-026-64170-y

**Published:** 2026-07-30

**Authors:** Jesenko Karačić, Sabina Karačić, Miriam Grosse, Andrea Schiefer, Kenneth Pfarr, Tanja Schneider, Achim Hoerauf, Gabriele Bierbaum

**Affiliations:** 1 Institute of Medical Microbiology, Immunology and Parasitology, University Hospital Bonn, University of Bonn, Venusberg-Campus 1, 53127 Bonn, Germany; 2 German Center for Infection Research (DZIF), Partner Site Bonn-Cologne, Bonn, Germany; 3 Department of Microbial Drugs, Helmholtz Centre for Infection Research, 38124 Braunschweig, Germany; 4 German Center for Infection Research (DZIF), Partner Site Braunschweig, Braunschweig, Germany; 5 Institute for Pharmaceutical Microbiology, University Hospital Bonn, University of Bonn, Meckenheimer Allee 168, 53115 Bonn, Germany

**Keywords:** Corallopyronin A, Staphylococci, Mutations, Rifampicin, Evolution, Genetics, Microbiology, Molecular biology

## Abstract

Corallopyronin A (CorA) is a natural RNA polymerase (RNAP) inhibitor active against Gram-positive pathogens, including *Staphylococcus aureus*. Although spontaneous resistance to CorA occurs less frequently than to rifampicin, the genetic basis and evolutionary constraints of CorA resistance remain incompletely defined. Spontaneous mutation frequencies to CorA and rifampicin were determined by fluctuation analysis in three *S. aureus* strains and two coagulase-negative staphylococci (CNS). Resistant mutants were phenotypically characterized and subjected to whole-genome sequencing. Double-resistant mutants were generated under rifampicin selection. Fitness costs were assessed by direct competition assays. Mutation frequencies to CorA were consistently lower than to rifampicin across all tested strains (Rif/CorA ratios 2.3–2.7). Whole-genome sequencing of 41 independently derived CorA-resistant mutants revealed resistance-associated substitutions confined to the RNAP subunits RpoB and RpoC. The mutational landscape was highly restricted, with recurrent hotspots at RpoC K334 and RpoB S1127. In coagulase-negative staphylococci (CNS), resistance remained RNAP-centred but displayed broader allelic diversity, including substitutions previously described in *S. aureus* but identified here for the first time in CNS species, as well as the previously undescribed RpoC D810Y substitution. Ten mutants with resistance to CorA and rifampicin retained the CorA-associated mutation and acquired additional rifampicin resistance substitutions at non-overlapping RNAP residues. CorA resistance imposed a moderate fitness cost (12–15%), while additional rifampicin resistance resulted in mutation-dependent increases in the fitness burden. CorA resistance in staphylococci arises at lower spontaneous frequencies than rifampicin resistance and is mediated by a constrained set of RNAP mutations. The restricted mutational landscape and associated fitness costs indicate that, under the in vitro conditions examined, CorA resistance arises through more constrained evolutionary pathways than rifampicin resistance. These findings provide a foundation for further evaluation of CorA as an RNA polymerase-targeting antimicrobial.

**Supplementary Information:**

The online version contains supplementary material available at 10.1038/s41598-026-64170-y.

## Introduction

Antibiotic resistance in *Staphylococcus aureus* and other staphylococci remains a major clinical concern, particularly in the setting of device-associated infections and the global spread of multidrug-resistant lineages^1–3^. Among essential bacterial targets, RNA polymerase (RNAP) represents a critical vulnerability, as its inhibition blocks transcription and rapidly impairs bacterial growth^4^. However, the clinical utility of the RNAP inhibitor rifampicin has been compromised by the rapid and predictable emergence of resistance, largely driven by point mutations in the β subunit encoded by *rpoB*^5,6^. These mutations arise at relatively high frequencies and readily expand under selective pressure, limiting sustained therapeutic efficacy^7,8^.

Corallopyronin A (CorA) is a natural α-pyrone antibiotic that inhibits bacterial RNAP through a mechanism distinct from that of rifampicin^7–9^. By interfering with transcription initiation at a different region of the RNAP complex^10,11^, CorA retains activity against diverse pathogens, including *S. aureus*, and remains active against isolates resistant to several other antibiotic classes^12–19^. Its distinct binding mode raises the possibility that resistance may evolve along alternative mutational routes within RNAP.

Although spontaneous resistance to CorA has been reported to occur at lower frequencies than resistance to rifampicin^15^, the mutational landscape and evolutionary constraints underlying CorA resistance remain incompletely defined^20,21^. It is unclear whether resistance arises through a broad spectrum of substitutions or is restricted to defined RNAP hotspots, whether similar determinants operate across different staphylococcal species, and how CorA resistance relates genetically to rifampicin resistance. In addition, the fitness consequences of CorA resistance and the potential for bacteria to accumulate resistance to both RNAP inhibitors have not been systematically examined.

Here, we investigated the genetic and evolutionary basis of CorA resistance in diverse staphylococcal backgrounds. We hypothesized that CorA resistance is mediated by a constrained set of RNAP mutations distinct from those selected by rifampicin and that accumulation of resistance to both agents may impose measurable fitness costs. To test these hypotheses, we combined fluctuation analysis, phenotypic characterization, and whole-genome sequencing of independently derived CorA-resistant mutants from multiple *S. aureus* lineages and coagulase-negative species, as well as double-resistant derivatives selected under rifampicin pressure.

## Material and method

### Bacterial strains

Seven staphylococcal strains were included in this study, comprising five *Staphylococcus aureus* strains (*S. aureus* USA300_FPR3757^22^, *S. aureus* 6850 ATCC 53657^23^, *S. aureus* Mu50^24^, *S. aureus* type strain ATCC 33591, and *S. aureus* HG001^25^) and two coagulase-negative staphylococci (CNS), *S. epidermidis* ATCC 14990 and *S. warneri* ATCC 27836. Genotypic characteristics of these strains have been described previously^26^.

Strains were stored at − 80 °C in glycerol stocks and grown overnight on Columbia blood agar at 37 °C before experimentation. For pre-cultures, single colonies were inoculated into Mueller-Hinton (MH) broth and incubated overnight at 37 °C with shaking (170 rpm) overnight. CorA-resistant mutants were generated as described below.

### Antibiotics

Corallopyronin A (CorA; purity > 90%) was produced at the Helmholtz Centre for Infection Research, Braunschweig, using a heterologous *Myxococcus xanthus* strain harbouring the complete CorA biosynthesis gene cluster and purified as previously described by Pogorevc et al. 2019^18^. Rifampicin was obtained from (Sigma-Aldrich, St. Louis, MO, USA) and prepared according to the manufacturer’s instructions.

### Antimicrobial susceptibility testing

The minimal inhibitory concentrations (MIC) of CorA and rifampicin were determined using the broth microdilution method according to EUCAST guidelines. Assays were carried out in sterile 96-well microplates with Mueller-Hinton (MH) broth (Oxoid, Thermo Scientific, Waltham, MA, USA) and an inoculum of 1–5 × 10^5^ CFU/ml. Plates were incubated at 37 °C for 24 h, and MICs were defined as the lowest concentration without visible growth. For CorA, the procedure followed a standardized protocol established in our previous study^26^. Briefly, stock solutions were prepared and stored at − 80 °C, and fresh working solutions (16 µg/ml MH, 2% DMSO) were prepared on the day of the assay. Twofold serial dilutions were performed across the plate, with appropriate growth and solvent controls (DMSO) included. Each assay was performed in triplicate, and results were confirmed in at least two independent experiments.

### Mutation frequency and rate analysis

Mutation frequencies were determined using 20 independent parallel cultures per strain. Cultures were grown in Iso-Sensitest broth at 37 °C with shaking until reaching an optical density OD_600_=1. For each culture, 1 mL was centrifuged (8000 rpm, 3 min), and pellets were resuspended in 100 µL broth. Aliquots (100 µL) were plated onto Iso-Sensitest agar containing either CorA or rifampicin at 4 × MIC, freshly prepared and protected from light. Serial tenfold dilutions were plated on non-selective Iso-Sensitest agar to determine total CFU counts. Plates were incubated at 37 °C for 24 h in the dark. Colony counts were used to calculate mutation frequencies and mutation rates. Mutation rates were calculated using the FALCOR online calculator (https://lianglab.brocku.ca/FALCOR/) with both the Lea-Coulson and MSS maximum likelihood methods. From each fluctuation assay, 5–10 resistant colonies per strain were randomly selected and purified on Iso-Sensitest agar containing the respective antibiotic. Colonies were grown overnight and stored at − 80 °C in MH broth supplemented with 20% glycerol. These mutant stocks were subsequently used for MIC determination, time-kill experiments, and whole-genome sequencing.

### Time-kill assays

Time-kill assays were performed with *S. aureus* HG001 and its CorA-resistant mutant harbouring K334N RpoC. Overnight cultures were diluted 1:100 into 40 mL of Mueller-Hinton (MH) broth and grown at 37 °C with shaking until mid-log phase (OD_600_=0.6). From these cultures, 15 mL were transferred into 150 mL Erlenmeyer flasks, and CorA was added at a final concentration of 4 × MIC. Cultures were incubated at 37 °C under constant agitation. Samples were collected at 0, 4, 8, 24, and 48 h after CorA exposure. Serial dilutions were plated on Columbia blood agar, incubated overnight at 37 °C, and viable counts were expressed as CFU/mL. Parallel control cultures without CorA were processed identically.

### Fitness cost determination

Relative fitness was determined by direct competition assays between resistant mutants and their corresponding parental strains, as previously described^6^.

Parental and mutant strains were grown independently to mid-log phase (OD_600_=0.6). Equal volumes (1 mL each) were mixed and diluted 10^−5^ into 5 mL of antibiotic-free Mueller-Hinton (MH) broth to establish the initial mixed culture (T₀). Serial dilutions of this culture were plated on MH agar with and without CorA (4 × MIC of the susceptible parent) to determine starting colony counts. Colony-forming units (CFU/mL) obtained on selective agar represented mutant counts (A_mut), whereas counts on non-selective agar represented total CFU (A_total). The parental strain counts (A_wt) were calculated as A_total − A_mut.

Mixed cultures were incubated at 37 °C with shaking for 20 h. After incubation (*T_final*), serial dilutions were plated again on selective and non-selective agar to determine final mutant (*B_mut*) and total (*B_total*) CFU counts. Final parental counts (*B_wt*) were calculated as *B_total* − *B_mut*. The number of generations (G) for mutant and parental strains during the competition interval was calculated according to:

G = (*log B* − *log A*) / *log 2*. Relative fitness (W) was calculated as: W = *G_mut* / *G_wt.*

Fitness cost (FC) was expressed as a percentage reduction relative to the parental strain: FC = (1 − W) × 100%. Experiments were performed in biological replicates.

### Whole-genome sequencing (WGS)

For whole-genome sequencing, CorA-resistant mutants were streaked on Iso-Sensitest agar supplemented with 4 × MIC CorA. Single colonies were inoculated into 50 mL tryptic soy broth (TSB) and incubated overnight at 37 °C with shaking. Cells were harvested (8000 × g, RT, 4 °C) for 15 min, and genomic DNA was extracted using the Zymo Research genomic DNA kit following the manufacturer’s protocol. Paired-end sequencing (2 × 150 bp) was performed on an Illumina NovaSeq platform (Azenta/Genewiz, Leipzig, Germany), yielding approximately 2 Gb of raw data per sample. Genomes were assembled *de novo* and aligned to the respective parental reference sequences using Geneious (version R10.2.6; https://www.geneious.com).

### Statistics and data analysis

Statistical analyses were conducted using RStudio (Version 2024.04.0 + 735, R Foundation for Statistical Computing, Vienna, Austria), with comparisons performed using Mann-Whitney U test. A p-value < 0.05 was considered statistically significant.

### Data availability

Raw whole-genome sequencing (WGS) reads have been deposited in the NCBI Sequence Read Archive (SRA) under accession PRJNA1449794.

## Results

### Spontaneous resistance to CorA emerges less frequently than to rifampicin

**Fig. 1:**
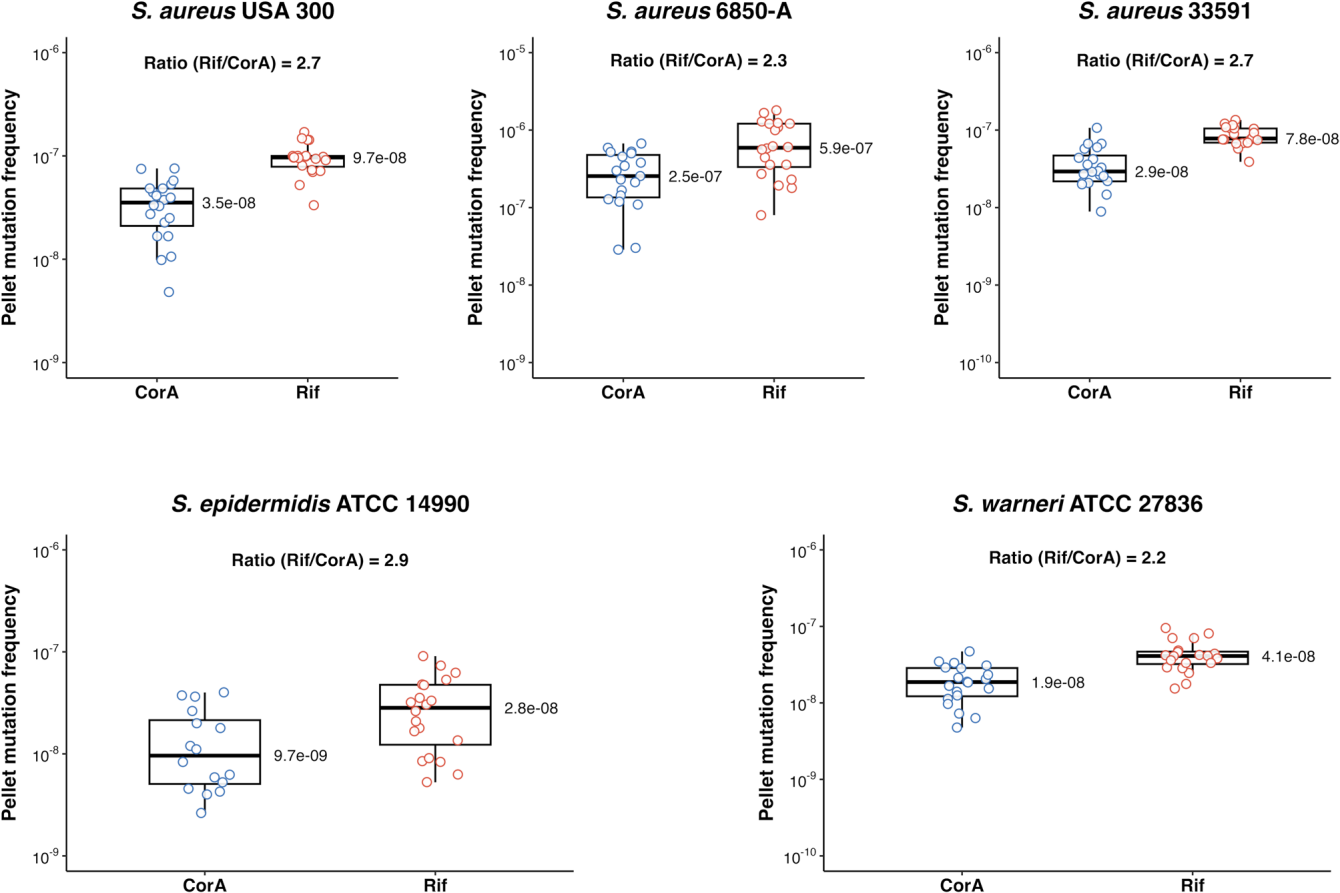
Frequency of mutation to CorA and rifampicin resistance in *S. aureus* and coagulase-negative staphylococci (CNS) strains. Box-and-whisker plots show pellet mutation frequencies obtained from 20 independent parallel cultures (*n* = 20) per strain on agar containing 4 × MIC of CorA or rifampicin. Each dot represents one independent culture. Boxes indicate the interquartile range (IQR), the horizontal line represents the median, and whiskers extend to 1.5 × IQR. Statistical comparisons between CorA and rifampicin mutation frequencies for each strain were performed using the Mann-Whitney U test.

To assess the frequency of spontaneous resistance development, three *S. aureus* strains were analysed using fluctuation assays with selection at 4 × MIC of either CorA or rifampicin. Mutation frequencies and mutation rates were determined for each strain (Fig. 1; Table S1).

Across all *S. aureus* strains, the median frequency of mutation (FoM) to CorA ranged from 2.4 × 10^−8^ to 3.6 × 10^−8^, whereas mutation frequencies to rifampicin were consistently higher (5.9 × 10^−8^ to 9.7 × 10^−8^). Statistical analysis using the Mann–Whitney U test confirmed significantly higher mutation frequencies for rifampicin compared with CorA in all strains examined (*S. aureus* USA300, *p* = 1.27 × 10_6; *S. aureus* 6850-A, *p* = 2.34 × 10_3; *S. aureus* ATCC 33591, *p* = 2.35 × 10_6).

The resulting Rif/CorA ratios ranged between 2.3 and 2.7, demonstrating a reproducible two- to three-fold greater likelihood of spontaneous resistance emergence to rifampicin under identical conditions. Similar results were obtained when mutation rates were calculated using both the Lea-Coulson and MSS maximum likelihood methods (Table S1).

To determine whether this pattern extends beyond *S. aureus*, two coagulase-negative staphylococci (CNS) type strains (*S. epidermidis* ATCC 14990 and *S. warneri* ATCC 27836) were analysed in parallel. Although absolute mutation frequencies in CNS were overall lower than in *S. aureus*, CorA mutation frequencies remained consistently below those observed for rifampicin. Median FoM values for CorA were in the range of 10^−9^ − 10^−8^, whereas rifampicin mutation frequencies were approximately two- to four-fold higher with statistically significant differences (*S. epidermidis* ATCC 14990, *p* = 0.0184; *S. warneri* ATCC 27836, *p* = 0.0065). The Rif/CorA ratios in CNS (2.2 and 2.9) were comparable to those observed in *S. aureus*, indicating that the reduced propensity for resistance development to CorA is conserved across staphylococcal species.

### CorA-mediated bactericidal activity is substantially reduced in resistant mutants

To confirm the resistance phenotype of colonies recovered from CorA-containing plates, MICs were determined for representative CorA-resistant mutants derived from all seven strain backgrounds. In all cases, mutants exhibited a pronounced increase in CorA MICs compared to their respective parental strains (Table 1). Depending on the strain, CorA MICs increased from baseline values of 0.25–0.5 µg/mL to levels ranging between 16 µg/mL and > 128 µg/mL.

Importantly, rifampicin MICs of CorA-resistant mutants remained unchanged relative to their parental strains, indicating that resistance to CorA does not confer cross-resistance to rifampicin. These findings support a target-specific mechanism of resistance rather than a generalized reduction in susceptibility.

**Table 1:** CorA and rifampicin MICs of parental strains and corresponding CorA-resistant mutants determined in Iso-Sensitest broth.

Strain	CorA		Rif
	MIC	MIC-mutant	MIC
*S. aureus* HG001	0.25	> 64	0.004
*S. aureus* Mu50	0.25	16/>128	0.004
*S. aureus* USA 300	0.25	> 64	0.004
*S. aureus* 6850-A	0.5	> 128	0.004
*S. aureus* ATCC 33591	0.5	> 64	0.004
*S. epidermidis* ATCC 14990	0.5	32/128	0.004
*S. warneri* ATCC 27836	0.5	16/>128	0.008

MIC values for CorA-resistant mutants represent independently derived isolates. Multiple values indicate distinct mutants with different levels of resistance. Rifampicin MICs are shown for the parental strains.

**Fig. 2:**
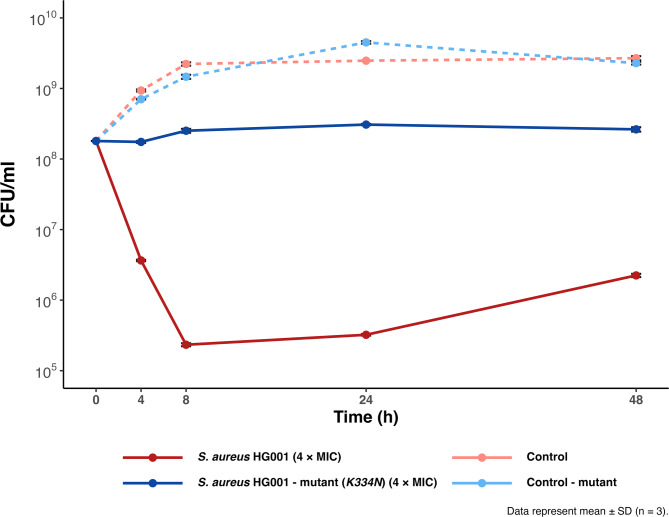
Time-kill curves of the parental *S. aureus* HG001 strain and its representative CorA-resistant mutant (RpoC K334N) exposed to CorA at 4 × MIC. The *S. aureus* HG001 (RpoC K334N) mutant was selected as a representative CorA-resistant mutant for time-kill analysis because K334N represented one of the most frequently observed CorA resistance substitution identified in this study. Viable counts (CFU/mL) were determined at the indicated time points. Untreated cultures served as controls. Data represent mean ± SD from three independent biological replicates (*n* = 3).

Time-kill assays were performed with *S. aureus* HG001 and its corresponding CorA-resistant mutant at 4 × MIC CorA. In the parental strain, CorA exposure resulted in rapid and pronounced bacterial killing, with an average reduction of ≥ 3 log_10_ CFU/mL relative to the initial inoculum (Fig. 2). Although partial regrowth was observed at later time points, CFU counts remained substantially reduced compared to untreated controls.

In contrast, the CorA-resistant mutant exhibited no significant reduction in CFU counts upon exposure to 4 × MIC CorA. Growth kinetics of the untreated mutant closely paralleled those of untreated controls throughout the experiment, indicating that acquisition of resistance effectively abolishes CorA-mediated bactericidal activity.

### Whole-genome sequencing reveals a constrained RNAP mutational landscape underlying CorA resistance

Whole-genome sequencing was performed for 41 independently derived CorA-resistant mutants obtained from seven staphylococcal strain backgrounds, together with their respective parental strains. Across all analysed isolates, resistance-associated mutations were confined to the RNA polymerase subunits *rpoB* and *rpoC*, consistent with a target-based mechanism of resistance.

**Fig. 3:**
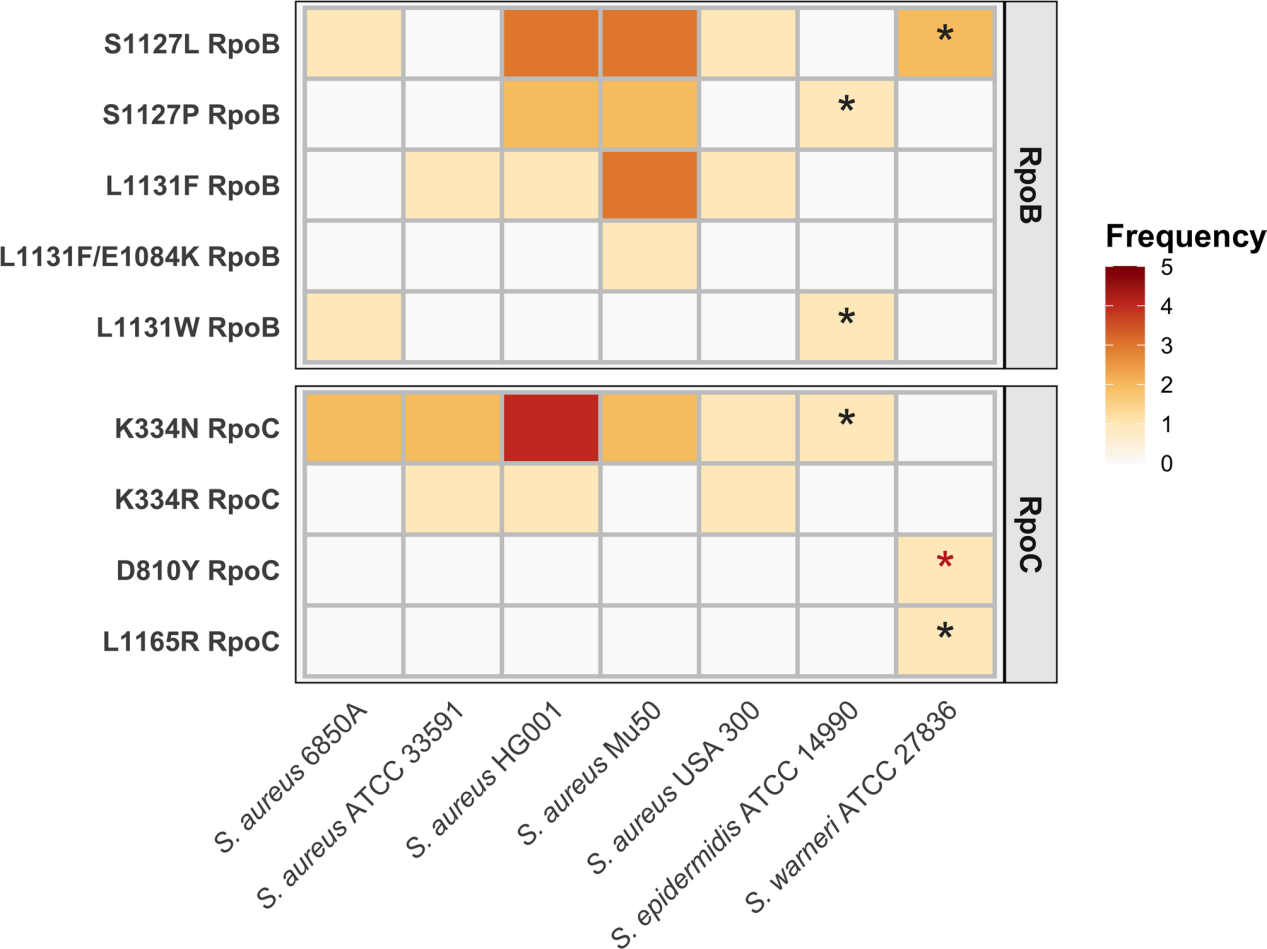
Distribution and frequency of amino acid substitutions identified among 41 independently isolated CorA-resistant mutants recovered from seven different staphylococcal strain backgrounds. The heatmap illustrates the number of mutants carrying each amino acid substitution in RpoB and RpoC. Color intensity corresponds to the frequency of the exchanges in the tested mutant strains. The red asterisk (*) denotes the previously undescribed RpoC D810Y substitution identified in *S. warneri*. Black asterisks (*) indicate substitutions identified for the first time in the tested coagulase-negative staphylococcal species, although previously reported in *S. aureus*.

The mutational spectrum was highly restricted, with substitutions repeatedly clustering at a limited number of RNAP positions (Fig. 3). In *S. aureus*, the most frequent amino acid exchange was RpoC K334N, detected across independent lineages and strain backgrounds. Recurrent substitutions were also observed at RpoB L1131 (L1131F/L1131W) and RpoB S1127 (S1127L/S1127P).

In contrast, the two coagulase-negative staphylococcal species exhibited broader allelic diversity while remaining restricted to RNAP. In *S. epidermidis*, resistance-associated substitutions included RpoB S1127P and RpoC K334N, which were also identified in *S. aureus*, together with RpoB L1131W, previously described in *S. aureus* but, to our knowledge, reported here for the first time in *S. epidermidis* (Fig. 3). In *S. warneri*, resistance-associated substitutions included RpoB S1127L and RpoC L1165R, both previously reported in CorA-resistant *S. aureus* mutants^29^ but identified here for the first time in *S. warneri*. Notably, we also identified the RpoC D810Y substitution in *S. warneri*, which, to our knowledge, has not previously been reported in any staphylococcal species and therefore represents a novel CorA resistance-associated substitution.

A complete list of resistance-associated substitutions identified across all species is provided in Supplementary Table S2.

### Combined CorA and rifampicin resistance remains genetically restricted to RNA polymerase

To evaluate the genetic compatibility of CorA and rifampicin resistance, CorA-resistant HG001 derivatives carrying the most frequent RpoC K334N substitution were subjected to rifampicin selection. Whole-genome sequencing was performed for ten independently derived double-resistant isolates (five per strain background).

All double mutants retained the CorA-associated RpoC K334N substitution and acquired additional exchanges exclusively in RpoB, consistent with rifampicin resistance. In strain HG001 8.2, identified substitutions included S486L, D471Y, Q137L, and R484H. In strain HG001 6.4, additional substitutions comprised S486L, Q137R, Q137L and L446S (Table 2).

Importantly, no recurrent mutations outside RNAP were detected, demonstrating that resistance to both agents is driven by distinct but compatible substitutions within RNA polymerase.

**Table 2:** Secondary rifampicin-resistance-associated RpoB substitutions identified in independently isolated *S. aureus* HG001 CorA-rifampicin double-resistant mutants.

*S. aureus* HG001			
Sample ID	CorA-resistant parent	RpoC (CorA-associated)	Additional RpoB (rifampicin-associated)
HG001 8.2_2	HG001 8.2	K334N	S486L
HG001 8.2_3	HG001 8.2	K334N	D471Y
HG001 8.2_5	HG001 8.2	K334N	D471Y
HG001 8.2_8	HG001 8.2	K334N	Q137L
HG001 8.2_10	HG001 8.2	K334N	R484H
HG001 6.4_1	HG001 6.4	K334N	S486L
HG001 6.4_4	HG001 6.4	K334N	Q137R
HG001 6.4_7	HG001 6.4	K334N	Q137L
HG001 6.4_6	HG001 6.4	K334N	L446S
HG001 6.4_9	HG001 6.4	K334N	S486L

Each row represents one independently isolated mutant and indicates the CorA-resistant parental background (*S. aureus* HG001 6.4 or *S. aureus* HG001 8.2), the parental RpoC K334N substitution, and the secondary RpoB substitution acquired following rifampicin selection.

### Fitness costs of CorA resistance and CorA-rifampicin double mutants

CorA resistance mediated by the recurrent RpoC K334N substitution was associated with a moderate but reproducible fitness cost across the analysed strain backgrounds. Direct competition assays performed in biological triplicates revealed fitness reductions of 12.43% in *S. aureus* strain HG001 6.4 and 16% in *S. aureus* strain HG001 8.2 relative to their respective parental strains, indicating that CorA resistance is not fitness-neutral (Table S4).

A comparable fitness burden was observed in *S. warneri*, where the CorA-resistant mutant carrying the same RpoC K334N substitution exhibited an average fitness cost of approximately 11–12% across triplicate competition assays. These results indicate that the recurrent RpoC K334N substitution was associated with a measurable fitness cost in the analysed *S. aureus* and *S. warneri* backgrounds.

To assess the biological consequences of combined RNA polymerase resistance, double-resistant mutants carrying additional substitutions in RpoB were analysed. These included the rifampicin-resistance alleles L446S and D471Y.

In strain HG001 6.4, the double mutant carrying RpoC K334N and RpoB L446S exhibited a slightly increased fitness cost (13.78 ± 0.38%), corresponding to only a modest increase relative to the CorA-only mutant (+ 1.35%).

In contrast, in strain HG001 8.2, the double mutant carrying RpoC K334N and RpoB D471Y displayed a higher average fitness cost (23.25 ± 2.0%) than the corresponding CorA-resistant parent (16%), although the limited number of biological replicates precluded robust statistical comparison.

Taken together, these findings suggest that while CorA resistance alone imposes a moderate fitness burden, the additional impact of rifampicin resistance depends strongly on the specific RpoB substitution rather than representing a uniformly additive effect. This observation suggests allele-specific interactions within the RNA polymerase complex, whereby particular combinations of RpoB and RpoC mutations differentially affect enzyme performance and bacterial fitness.

## Discussion

The present study provides a comprehensive analysis of the genetic and evolutionary basis of resistance to CorA in staphylococci. Across seven strain backgrounds and 41 independently derived mutants, resistance was consistently mediated by mutations in the RNA polymerase subunits RpoB and RpoC, with substitutions clustering at a limited number of recurrent positions. These findings indicate that CorA resistance is strongly target-restricted and follows a reproducible mutational trajectory which mainly includes the amino acids that bind CorA or that lie directly adjacent to amino acids binding CorA^11,27^.

Resistance to rifampicin is well documented to arise at high frequencies through diverse substitutions within defined regions of RpoB, particularly within the rifampicin resistance - determining region (RRDR)^6,19,28^. Previous studies have reported a broad mutational spectrum associated with rifampicin resistance; for example, *Wichelhaus et al.*. 2002 identified 24 distinct RpoB substitutions among 54 resistant *S. aureus* isolates. In contrast, we observed that spontaneous resistance to CorA emerged at lower frequencies and was associated with a comparatively narrow set of RNAP substitutions, with nine distinct mutations identified among 41 independently derived mutants^6^. Seven of the resistance-associated amino acid substitutions identified in the present study were previously described by Mariner et al.^29^ and Balansky et al.^15^. This restricted mutational spectrum suggests that the structural interface targeted by CorA may tolerate fewer adaptive alterations, with only a limited number of binding-site residues that can be modified without compromising enzymatic activity.

The spontaneous mutation frequencies reported in the present study were obtained to provide a broader data set for the findings on mutation frequency in the presence of CorA using more *S. aureus* strains and including CNS as well as to generate CorA-resistant mutants for whole-genome sequencing and downstream phenotypic characterization. Accordingly, the quantitative Rif/CorA mutation frequency ratios differ modestly from that reported previously by Balansky et al.^15^, which was determined only for strain *S. aureus* HG001, although the overall conclusion remained unchanged. In both studies, spontaneous resistance to CorA arose at substantially lower frequencies than resistance to rifampicin, supporting a restricted mutational pathway to CorA resistance. The observed differences most likely reflect biological variation between different strains rather than methodological differences.

A similar principle of constrained mutational accessibility has been described for other RNAP inhibitors targeting the RNAP switch region, including myxopyronins, which also select for defined substitutions in the RNA polymerase genes *rpoB* and *rpoC*, resulting in amino acid substitutions that clustered at a limited number of recurrent positions^11,27^. Structural studies have identified key contact residues in both RpoB and RpoC that contribute to inhibitor binding in the *E. coli* RNA polymerase^11^. Notably, several of the substitutions identified in the present study map to positions corresponding to, or located in proximity to, these residues. For example, the recurrent RpoC K334 substitution corresponds to K345 in *E. coli* RNAP, a residue directly implicated in inhibitor interaction. These observations support the notion that CorA resistance primarily arises through amino acid exchanges of the RNAP switch region that inhibit binding of CorA. However, given the limited number of mutants analysed, additional resistance-associated substitutions may exist that were not captured in the present dataset.

The identification of the previously undescribed RpoC D810Y substitution in *S. warneri*, together with CorA resistance-associated substitutions previously reported in *S. aureus* but identified here for the first time in coagulase-negative staphylococci, further expands the spectrum of RNAP mutations associated with CorA exposure. Residue D810 is one of the residues that was previously determined to mediate contact to the sidechain of CorA via a water molecule^9^ and, therefore, substitution of the negatively charged aspartate will impede binding of CorA. Because the surrounding RpoC sequence is highly conserved among the investigated staphylococcal species, the emergence of D810Y is unlikely to reflect species-specific sequence variation but rather represents an additional resistance-conferring alteration within the conserved CorA-binding region.

Importantly, acquisition of rifampicin resistance in CorA-resistant strains resulted in non-overlapping substitutions within RNAP, demonstrating that resistance to both inhibitors can be accommodated within the same target. Nevertheless, fitness analyses revealed mutation-dependent biological consequences. Similar results were also obtained for myxopyronin resistant mutants^27^. While CorA resistance alone imposed a moderate fitness cost, the additional burden of rifampicin resistance varied depending on the specific RpoB substitution. Similar variability has been described for rifampicin resistance mutations, where different RpoB substitutions can impose markedly different fitness effects, ranging from substantial growth impairment to nearly neutral effects. Together, these findings suggest that although resistance to two mechanistically distinct RNAP inhibitors can co-occur, the accumulation of certain mutation combinations may be constrained by mutation-specific fitness trade-offs. Although the exact mutation frequency could not be determined, the previously reported observation that no resistant mutants were recovered after plating >10¹¹ bacterial cells on agar containing rifampicin and CorA (Balansky et al., 2022) ^15^ is consistent with our findings and further supports the potential of this combination to limit the emergence of resistance.

Although our findings were obtained under in vitro conditions, they provide several observations that may be relevant for the future development of RNA polymerase-targeting antibiotics. The lower spontaneous mutation frequency observed for CorA compared with rifampicin likely reflects the limited number of RNAP residues that can confer resistance when altered, thereby reducing the probability of resistance emergence under antibiotic pressure. In addition, the measurable fitness costs associated with both single and combined RNAP resistance mutations may influence the persistence and dissemination of resistant clones if similar fitness effects occur in vivo. Together, these features support further evaluation of CorA as a potential therapeutic alternative or complement to existing RNAP inhibitors in the treatment of multidrug-resistant staphylococcal infections.

This study has limitations. Resistance evolution was assessed under in vitro conditions, and the stability of identified mutations in the absence of antibiotic pressure was not systematically examined. In addition, structural modelling of resistance-associated substitutions was not performed. Whole-genome sequencing consistently identified mutations in *rpoB* and *rpoC* as the only recurrent genetic changes associated with CorA resistance. However, genetic complementation or allelic replacement experiments were not performed to directly confirm the individual contribution of each mutation to the resistant phenotype. Because all experiments were performed under laboratory conditions, the observed mutation frequencies, evolutionary trajectories, and fitness effects may not fully reflect resistance evolution in vivo. Future studies integrating structural and in vivo approaches will be required to fully define the mechanistic and clinical implications of CorA resistance.

In conclusion, CorA resistance in staphylococci is mediated by a constrained set of RNA polymerase mutations and arises at lower spontaneous frequencies than rifampicin resistance. The restricted mutational landscape, compatibility of dual RNAP resistance, and mutation-dependent fitness costs provide new insights into the evolutionary dynamics of resistance to this transcriptional inhibitor. As these findings are based on in vitro experiments, further validation in vivo will be required to establish their clinical relevance.

## Supplementary Information

Below is the link to the electronic supplementary material.

Supplementary Material 1

Supplementary Material 2

## Data Availability

Raw whole-genome sequencing (WGS) reads have been deposited in the NCBI Sequence Read Archive (SRA) under accession PRJNA1449794.
