# Supplementary material for: Constrained RNA polymerase mutational pathways distinguish corallopyronin A resistance from rifampicin resistance in staphylococci
Source: Sci Rep. 2026 Jul 30;16:23649. doi: 10.1038/s41598-026-64170-y (PMC13424638; doi:10.1038/s41598-026-64170-y)
Supplement: Supplementary file 1 — Supplementary Material 1 [file 41598_2026_64170_MOESM1_ESM.docx]

**Supplementary Table S1:** Mutation frequencies and mutation rates associated with CorA and rifampicin resistance across staphylococcal strain backgrounds. Median frequency of mutation (FoM), mutation rates estimated by the Lea-Coulson (LC) method, and mutation rates estimated by the Ma-Sandri-Sarkar (MMS) maximum likelihood method are shown. Values are reported for CorA and rifampicin selection, together with the corresponding Rif/CorA ratios.

***S. aureus* USA300_FPR3757**

|  | **CorA** | **Rif** | **Rif/CorA** |
| --- | --- | --- | --- |
| Median of FoM | 3.55E-08 | 9.66E-08 | 2.73 |
| Mutation rate (LC) | 1.33E-08 | 2.71E-08 | 2.04 |
| Mutation rate (MMS) | 1.26E-08 | 2.99E-08 | 2.37 |

***S. aureus* 6850 ATCC 53657**

|  | **CorA** | **Rif** | **Rif/CorA** |
| --- | --- | --- | --- |
| Median of FoM | 2.43E-08 | 5.92E-08 | 2.44 |
| Mutation rate (LC) | 9.26E-09 | 1.70E-08 | 1.84 |
| Mutation rate (MMS) | 8.66E-09 | 1.72E-08 | 1.99 |

***S. aureus* type strain ATCC 33591**

|  | **CorA** | **Rif** | **Rif/CorA** |
| --- | --- | --- | --- |
| Median of FoM | 2.9E-08 | 7.80E-08 | 2.66 |
| Mutation rate (LC) | 1.00E-08 | 2.30E-08 | 2.30 |
| Mutation rate (MMS) | 1.20E-08 | 2.62E-08 | 2.18 |

***S. epidermidis* ATCC 14990**

|  | **CorA** | **Rif** | **Rif/CorA** |
| --- | --- | --- | --- |
| Median of FoM | 9.70E−09 | 2.80E-08 | 2.90 |
| Mutation rate (LC) | 2.96E-09 | 9.32E-09 | 3.15 |
| Mutation rate (MMS) | 3.08E-09 | 9.61E-09 | 3.12 |

***S. warneri* ATCC 27836**

|  | **CorA** | **Rif** | **Rif/CorA** |
| --- | --- | --- | --- |
| Median of FoM | 1.90E-08 | 4.10E-08 | 2.20 |
| Mutation rate (LC) | 5.28-09 | 1.16E-08 | 2.20 |
| Mutation rate (MMS) | 5.64E-09 | 1.22E-08 | 2.16 |
